# Research on the Optimization of Dietary Energy Supply in Growing and Fattening Pigs Under a Low-Temperature Environment

**DOI:** 10.3390/ani15081117

**Published:** 2025-04-12

**Authors:** Yu Zhang, Zhaoyang Qi, Guixin Qin, Hailong Jiang, Rui Han, Dongsheng Che

**Affiliations:** Jilin Provincial Swine Industry Technical Innovation Center, Jilin Provincial Key Laboratory of Animal Nutrition and Feed Science, Ministry of Education Laboratory of Animal Production and Security, College of Animal Science and Technology, Jilin Agricultural University, Changchun 130118, China; 18943098852@163.com (Y.Z.); 18943117305@163.com (Z.Q.); qgx@jlau.edu.cn (G.Q.); hljiang2024@163.com (H.J.)

**Keywords:** energy supply optimization, energy level, energy metabolism, growth performance

## Abstract

The purpose of this study was to investigate the effects of the optimization of dietary energy supply on the growth performance, nutrient digestibility, energy metabolism, nutrient oxidation, slaughter performance, and meat quality of growing and fattening pigs under a low-temperature environment. In this study, forty-eight 60-day-old growing barrows (Duroc × Landrace × Large White) with an initial body weight of 31.24 ± 3.56 kg were completely randomized into two treatment groups, with four replicates in each treatment group and six pigs in each replicate. The two groups were fed diets with equal protein levels and different energy levels (a conventional diet and an energy-optimized diet); the dietary energy level was increased by 8% by adding 6% fat, and the two groups were kept at the same ambient temperature (10 ± 1 °C) all day. After 5 d of prefeeding, the final weight reached approximately 110.00 kg prior to slaughter (99 days of age), and four pigs with a body weight of about 80.00 kg were selected in the two groups for digestion, metabolism, and respiratory calorimetry. The results showed that the average daily feed intake of the TES group (energy-optimized diet group, high fat and energy) was lower than that of the CON group (conventional diet group, normal fat and energy) (*p* < 0.05). Compared with the CON group, the feed-to-gain ratio was lower in the TES group during the fattening period (60–110 kg) (*p* < 0.05). Compared to the CON group, fat and energy digestibility in the TES group were higher (*p* < 0.05), fecal nitrogen and urine nitrogen were lower (*p* < 0.05), the nitrogen deposition rate increased (*p* < 0.05), and fat oxidation and the sedimentation energy rate also increased (*p* < 0.05). The serum triglyceride concentration in the TES group was higher than that in the CON group (*p* < 0.05). Compared to the CON group, the carcass weight, body fat content, backfat thickness, and eye muscle area in the TES group increased (*p* < 0.05); the L* value of flesh color also increased (*p* < 0.05); and the shear force was lower (*p* < 0.05). The dietary energy should be optimized under a low-temperature environment, and the feed conversion efficiency of fattening pigs could be improved by improving dietary energy levels by adding fat, increasing the fat oxidation proportion, promoting nitrogen deposition and sedimentation energy, and improving slaughter performance and meat quality.

## Simple Summary

Energy distribution in the diet of pigs is important because of the effects of differences in their dietary energy levels under a low-temperature environment. Therefore, we conducted a study to determine the growth performance, nutrient digestibility, and energy metabolism of fattening pigs and to optimize their dietary energy nutrient programs. At the same time, we used these indicators to provide reasonable recommendations for pig dietary energy nutrition systems.

## 1. Introduction

In recent years, the pig industry has gradually shifted to North China. Due to the geographical location of the northern region, the average daily temperature is below the lower limit of the optimal temperature for pigs (18 °C) for about 8 months of the year, with an average annual temperature of only 7.6 °C [1]. Moreover, based on the characteristics of cold regions in northern China and contemporary research records concerning regional pastures, it can be inferred that the indoor temperature during winter is approximately 10 °C when heating is not utilized [2]. The digestion, absorption, and metabolism of energy in animals are affected by the ambient temperature. When the ambient temperature is lower than the optimal temperature for pigs, the energy demand increases because body heat production increases under a low-temperature environment, and to maintain body temperature, they need to consume more energy. Studies have reported that in the temperature range of 5~20 °C, the metabolizable energy intake [3] and heat production [4,5,6] increase linearly with decreasing ambient temperature. When the temperature decreases by 1 °C, the caloric production per kilogram of metabolizable body weight of growing pigs increases by approximately 15.48~18.83 kJ/d [7]. Therefore, it is necessary to increase the dietary energy level appropriately to compensate for the energy loss caused by the increase in heat production to a certain extent under a low-temperature environment.

When the body uses ingested energy sources to produce heat, there is a certain sequence in the utilization of different energy carrier substances (carbohydrates, fat, protein). In general, fat and carbohydrates are the main energy sources, and when glucose oxidation cannot meet the body’s energy needs, fat is needed for oxidation [8]. Previous studies have shown that the average daily feed intake, average daily gain, digestibility of dietary nutrients, and feed conversion efficiency of piglets decrease with a decrease in the energy value ratio of carbohydrate to fat [9], which may be due to the high amount of fat added, which exceeds the ability of piglets to digest fat. However, the body energy demand changes to a certain extent; there is enough fat to be oxidized and prioritized, and the conversion efficiency is higher under a low-temperature environment [10]. For example, when growing pigs are fed high-fat diet, 10~20% fat oxidation occurs, while the contribution rate of carbohydrate oxidation to heat production decreases, and when carbohydrate and fat oxidation cannot meet the body’s needs, protein oxidation increases [11,12]. Therefore, various energy carrier substances ingested by pigs or deposited in body tissues can also be used to “burn” heat production to maintain body temperature. Under the premise of restricting feeding, Zhou et al. showed that the addition of soybean oil to increase energy levels was beneficial to improve energy utilization efficiency and reduce greenhouse gas CO_2_ emissions in Songliao black fattening pigs, and these changes were more pronounced in cold environments [12]. The efficiency and economic benefits of using different energy sources are different. For example, the energy released by complete oxidation per kilogram of fat in the body is approximately 2.25 times greater than that of carbohydrates. When fat and carbohydrates are insufficient, the body uses protein to produce heat, resulting an economic cost and nitrogen emission increase. Therefore, regardless of the results of analyses of the energy metabolism of pigs or the economic benefits of pig production, energy nutrition in cold areas must be optimized.

Most of the relevant studies on improving the dietary energy levels of animals under a low-temperature environment do not consider the composition of energy sources or energy supply efficiency and use only a single “energy value” to explain the energy and nutritional needs of animals, ignoring the large amount of energy loss accompanied by the mutual conversion of different energy sources in animals [13]. This experiment was based on a large number of studies at home and abroad, considering the different energy sources in the diet and the environmental temperature, and the dietary energy was further optimized and compared with the traditional diet. It was ensured that the protein and trace element intake of the diet were the same to investigate the effects of improving dietary energy levels by adding fat to the diet on the growth performance, nutrient digestibility, energy metabolism, oxidation of various nutrients, carcass traits, and meat quality of fattening pigs and thus provide a theoretical basis for optimizing the energy nutrition systems of fattening pigs in cold areas.

## 2. Materials and Methods

### 2.1. Experimental Design and Diets

In this study, forty-eight 60-day-old growing barrows (Duroc × Landrace × Large White) with an initial weight of 31.24 ± 3.56 kg were randomly divided into 2 treatment groups (a control group (CON, conventional diet) and a test group (TES, energy-optimized diet)). The ambient temperature was kept at 10 ± 1 °C, and the 2 groups were fed diets with an equal digestible crude protein level, with each group containing 4 replicates and each replicate 6 pigs. After 5 d of prefeeding, the formal feeding experiment began, the final weight reached approximately 110.00 kg prior to slaughter (99 days of age). The CON diet adhered to the NRC (2012) [14] nutritional requirements for growing and fattening pigs, the preparation of the diets in the growing stage (30–60 kg, and the duration of this trial was 43 days) and fattening stage (60–110 kg, and the duration of this trial was 56 days), and the CON group contained a fat content of 3.15% with a digestible energy level of 14.20 MJ/kg; conversely, the TES group contained a fat content of 8.09% along with a digestible energy level of 15.34 MJ/kg. The dietary formulations maintained uniform levels of digestible crude protein and amino acids. The diet was modified at the conclusion of the growth phase. In the fattening stage, the CON group contained a fat content of 3.69%, resulting in a digestible energy level of 14.02 MJ/kg; meanwhile, the TES group maintained 8.33% fat alongside a digestible energy level of 15.14 MJ/kg, as detailed in the chemical composition analysis presented in Table 1.

### 2.2. Animal Management

All experimental procedures in this study were performed in accordance with the guidelines for the management and use of laboratory animals at Jilin Agricultural University (Changchun, China). This animal experiment was approved by the Ethics Committee of Jilin Agricultural University and conducted at the Animal Husbandry Branch of the Jilin Academy of Agricultural Sciences (Changchun, China).

The feeding test was carried out at the experimental base of the Animal Husbandry Branch of the Jilin Academy of Agricultural Sciences. During the experiment, each pig had a certain feed intake and drank water freely throughout the whole process every day, and the feeding amount was adjusted daily according to the previous day’s feed intake. The building was naturally ventilated. The enclosure was washed daily and disinfected regularly to keep the enclosure clean, dry, and hygienic. The temperature of the enclosure was also kept at 10 °C, and the relative humidity was set to 70%. During the test, the health of the test pigs was observed and recorded every day. Four pigs with a body weight of about 80.00 kg were selected in the two groups and transferred to open-circuit respiration chambers.

### 2.3. Measurement Indicators and Methods

#### 2.3.1. Growth Performance

At the beginning and end of the experiment, the pigs were weighed in two stages (the growing and fattening stages) after 12 h of fasting (all animals had ad libitum access to feed and water), and the values were taken as the initial body weight (IBW) and final body weight (FBW), respectively. During the experiment, feed consumption was recorded for each pig, and the average daily feed intake (ADFI), average daily gain (ADG), and feed/weight ratio (FCR) were calculated separately during the growing and fattening stages.

The growth performance indicators were calculated as follows:

ADG = (FBW − IBW)/the number of days in the experiment.

ADFI = feed consumption/the number of days in the experiment.

FCR = feed consumption/total weight gain.

#### 2.3.2. Nutrient Digestibility and Nitrogen Balance

The digestion and metabolism tests were carried out in the open-circuit respiratory chambers of the Animal Husbandry Branch of the Jilin Academy of Agricultural Sciences, each of which has a single chamber. Each chamber can detect the indoor temperature and humidity through temperature and humidity sensors, and the temperature and humidity are individually controlled by high-precision devices such as gas circulation, refrigeration, and dehumidification devices. The indoor temperature is set separately according to the test requirements, and the humidity is kept at 70%. One week before the test, each feeding chamber, indoor metabolizable cage, and feed and water trough was thoroughly cleaned and disinfected. Regular feeding was carried out at 8:00 a.m. and 5:00 p.m. every day, and the pigs were allowed to feed and drink freely.

In this study, digestion and metabolism tests were performed via the total fecal urine collection method. Every day before early feeding, feces and urine were collected from the previous 24 h, the amount of fecal urine excreted and the date were recorded. The fecal urine samples collected daily were mixed well, and then, 200 g and 200 mL were removed from the samples, fixed with 10% sulfate and nitrogen, respectively, and stored at −20 °C for subsequent analyses. The prefeeding period was 3 d, and all of the samples were mixed after collection for 5 d. The fecal sample was dried in a 65 °C oven before the composition of the substance was determined and then crushed and sieved before preservation, and the urine was filtered through gauze to prevent other impurities from interfering with the measurement results. In compliance with the guidelines of the Chinese national standards, the content of crude protein (CP) and ether extract (EE) in the diet, feces, and urine was accurately measured. Additionally, to ascertain the gross energy (GE) present in the diet, feces, and urine, an isoperibol calorimeter (Parr 6300 Calorimeter, Moline, IL, USA) was utilized, employing benzoic acid as a calibration standard. These analyses were systematically replicated three times to ensure precision and reliability. The main calculations were as follows:

Fat digestibility (%) = (total fat intake − total fecal fat) ÷ total fat intake × 100;

Crude protein digestibility (%) = (total crude protein intake − total fecal crude protein) ÷ total crude protein intake × 100;

Energy digestibility (%) = (total feed energy intake − total fecal energy) ÷ total feed energy intake × 100;

Fecal nitrogen (FN, g/d) = average daily fecal weight × fecal nitrogen content;

Urine nitrogen (UN, g/d) = average daily urine volume × urine nitrogen content;

Nitrogen intake (NI, g/d) = average daily feed intake × feed nitrogen content;

Deposited nitrogen (ND, g/d) = NI − FN − UN;

Nitrogen deposition rate (NDR, %) = (ND/NI) × 100.

#### 2.3.3. Energy Metabolism

In this test, an 8-open-circuit respiration chamber inhalation and exhalation and heat measurement system was used for gas collection, and the net volume in the equipment was 3.2 m^3^. Before each test, the analyzer was calibrated with standard gas. Tests were carried out after 5 d of cleaning, disinfection, and ventilation with a respiratory thermometer. The pigs that had reached approximately 80.00 kg were pushed into the open-circuit respiration chambers. The glass doors were closed to prevent gas leakage, and the recovery rate was reduced. Each respiration chamber could measure the gas production and emission of only 1 pig. The experiment started after 1 week of adaptation to the environment and the diet, and all the data were recorded for 5 d. The respiration chamber gas analyzer first collected O_2_ and CO_2_ outside and O_2_ and CO_2_ in the eight metabolic chambers in order, and it took 3 min to collect gas in each small chamber. After collection, the system automatically calculated the CO_2_ produced and O_2_ consumed according to the difference between the indoor and outdoor O_2_ and CO_2_ amounts, cycled once every 27 min, and automatically saved the test results.

The total heat production (HP) was calculated via Brouwer’s formula as follows [19]:

HP (kJ) = 16.18 × O_2_ (L) + 5.02 × CO_2_ (L) − 2.17 × CH_4_ (L) − 5.99 × UN (g)

Since the amount of methane produced by pigs that consume these feeds is very low, the methane energy of the pigs can be ignored [20]. On the basis of nitrogen balance, the energy (RPE, kJ) in the protein form was calculated as UN × 6.25 × 23.86 kJ/g, and the difference between the energy in the form of sedimentation energy and RPE in the form of fat (RFE, kJ) was calculated [21]. The respiratory quotient (RQ) is the ratio of CO_2_ production to O_2_ consumption.

Chwalibog et al. described the calculation of OXPRO, OXCHO, and OXFAT [22]. The specific formula is as follows:

OXPRO (kJ) = UN (g) × 6.25 × 18.42 (kJ), OXCHO (kJ) = [−2.968 × O_2_ + 4.174 × CO_2_ − 1.761 × CH_4_ − 2.446 × UN (g)] × 17.58, OXFAT (kJ) = [1.719 × O_2_ − 1.719 × CO_2_ − 1.719 × CH_4_ − 1.963 × UN (g)] × 39.76.

#### 2.3.4. Serum Biochemical Indicators

In the early morning of the last day of the feeding test, each pig was collected on an empty stomach, and a blood sample was collected from the jugular veins of the pigs in the large pen using a syringe. The mixture was placed in a 10 mL serum separator tube, allowed to stand for 0.5 h, and centrifuged at 4 °C at 799× *g* for 15 min by using a 5430 Eppendorf machine (Hamburg, Germany), after which the supernatant was divided into aliquots and stored in a −80 °C freezer. The serum biochemical indices used in this test were as follows (automatic biochemical instrument, BS-400, Mindray, Shenzhen, China): serum urea nitrogen (BUN), total protein (TP), blood glucose (GLU), triglyceride (TG), high-density lipoprotein (HDL), low-density lipoprotein (LDL), aspartate aminotransferase (AST), alanine aminotransferase (ALT).

#### 2.3.5. Slaughter Performance and Meat Quality

After 12 h of feeding on the last day of the experiment, all pigs were slaughtered. The pigs were slaughtered and the carcass weight, dressing percentage, backfat thickness, and loin eye muscle area were measured according to the “Technical Specification for the Determination of Carcass Traits of Lean Pigs” (NY/T825-2004) [23], and the amount of lean meat [24], body fat content [25], lean meat percentage, and body fat percentage were calculated.

Carcass weight (kg): After the fattening pig was slaughtered and shaved, the head, hooves, tail, and internal organs were removed, and the body weight of the plate oil and kidneys was retained.

Pre-slaughter live weight (kg): The body weight after 12 h of feeding before slaughter.

Dressing percentage (%) = Carcass weight/pre-slaughter weight.

Backfat thickness: The 4 cm from the dorsal midline of the posterior edge of the scapula, the last rib, and the lumbar junction were used as the measurement points of fat thickness and eye muscle thickness, and then, the average of the three points was taken.

Eye muscle area: the cross-sectional area of the longissimus dorsal lumbar muscle between the penultimate first and second thoracic vertebrae of the pig.

*Y* = −6.9144 + 0.6154*X*_1_ − 2.6893*X*_2_.

*Y* is the lean meat content (kg); *X*_1_ is the live weight before slaughter (kg); *X*_2_ is the thickness of the backfat (cm) of the 6th–7th intercostal space.

*W* = − 26.4 + 0.221*EBW* + 1.331*P*_2_.

*W* is the body fat content (kg); *EBW* is the empty live body weight (kg), *EBW* = 0.905 × BW^1.013^; BW is the live body weight (kg); *P*_2_ is the backfat thickness: 6.5 cm from the dorsal midline at the last rib of the right half of the pig’s carcass.

Lean meat percentage (%) = lean meat content/pre-slaughter weight.

Body fat percentage (%) = body fat content/pre-slaughter weight.

The left half-carcass of each pig was suspended. An incision was made starting from the anterior end of the third thoracic vertebra (counting caudally) and extended posteriorly until the longissimus thoracis was isolated to meet the specified measurement criteria for length and weight. Meat quality parameters were determined as previously described [26,27]. The specific determination methods were as follows:

pH_24 h_: The pH meter was calibrated before the measurement, and pH 4.00 and pH 6.86 standard solutions were used for calibration. We took the longissimus dorsi muscle of the penultimate to second thoracic vertebrae, removed the fleshy peripheral muscle membrane, inserted the pH meter electrode probe into the cross so that the muscle completely wrapped around the electrode probe, and the measurement was carried out until the reading was stable Three different measurement points were measured in the same meat sample, and the results are expressed as the average value. The resulting muscle pH was measured 24 h after slaughter and labeled as pH_24_.

Drip loss: On the middle part of the longissimus dorsi muscle, we peeled off the fascia and fat on the surface, cut the sample meat slice with a thickness of about 2.5 cm along the direction of the muscle fiber, put the sample into a drip loss measurement tube along the direction of the muscle fiber, recorded its number, and put it in the refrigerator at 2~4 °C. When the time of the sample reached 48 h, we took out the sample and used filter paper to absorb the residual liquid on the surface of the sample (without squeezing or pressing), and then weighed the sample.

Cooking loss: Within 24 h after slaughter, a 2.5 cm thick meat sample was taken from the longest dorsal muscle, and the fat and epimysium were trimmed and weighed. Then, we placed the meat sample in a polyethylene plastic bag, removed the air from the bag, and sealed it, making sure that the surface of the meat sample was close to the plastic bag. The sealed meat sample bag was kept in a 75 °C water bath for 30 min, and the meat sample bag after the water bath was cooled under 15 °C running water for 40 min, and then, the plastic bag was opened and the moisture on the surface of the meat sample was wiped off with filter paper and the meat was weighed.

Shear force: From the longest lumbar dorsal muscle sample, we removed the surface fascia and fat, put it into a ziplock bag for maturation (after storage at 15 °C for 24 h, transfer to the refrigerator at 4 °C for storage for 48 h), and took it out after the maturation was completed. We inserted a thermometer into the center of the meat sample, tied the bag mouth tightly, laid it flat at room temperature for a period of time, placed it in a constant-temperature water bath at 70–80 °C, and took it out after continuous heating until the meat sample temperature reached 70 °C; then, we took it out until it reached cold to room temperature. Cuboid meat pieces with a parallel direction of the muscle fiber, a cross-sectional area of 1 cm^2^, and a length of 4~5 cm were cut, and the shear force value of ten meat pieces was measured by a tenderness analyzer; the average value was taken, and the unit was expressed in N.

Marbling score: The longissimus dorsi muscle at the thoracolumbar junction was taken and the meat sample was divided into two parts and placed on a white disc. The sample and a flesh color palette were compared under natural light for visual scoring, and the color was scored on a 6-point scale: 1 was PSE meat (light reddish-white to white); 2 is mild PSE meat (light gray-red); 3 is normal flesh color (bright red); 4 is normal flesh color (dark red); 5 is mild DFD meat (light purplish-red); 6 is DFD meat (deep purplish red).

Meat color: We used a calibrated colorimeter to determine the flesh color, removed fascia and fat from the surface, and tried to avoid bruises when determining the flesh color value. Measurements were taken three times in a row at different locations and the average was finally taken.

### 2.4. Statistical Analysis

After the preliminary summary statistics of the experimental data were obtained via Excel 2007, an independent sample T-test analysis was carried out on the data via SPSS 23 (IBM-SPSS Inc., Chicago, IL, USA). The means ± SEM were reported, with statistical significance set at *p* < 0.05.

## 3. Results

### 3.1. Growth Performance

The TES group had a higher FBW in the fattening stage than the CON group (Table 2, *p* < 0.05), and the ADFI in the growing and fattening stages decreased (*p* < 0.01). The FCR decreased by 16.15% during the fattening stage (*p* < 0.05). There was no difference in FCR during the growth stage between the TES and CON groups (*p* > 0.05). There was no difference in ADG between the two groups, but that in the TES group was higher than that in the CON group (*p* > 0.05).

### 3.2. Nutrient Digestibility and Nitrogen Desposit

Compared to the CON group, the EE and energy digestibility in the TES group increased (Table 3, *p* < 0.01), but there was no effect on CP digestibility (*p* > 0.05). In terms of nitrogen balance, FN and UN in the TES group were lower than those in the CON group (*p* < 0.05). The nitrogen deposition rate increased (*p* < 0.05). There was no difference in nitrogen deposition between the two groups, but nitrogen intake in the TES group was lower than that in the CON group (*p* > 0.05).

### 3.3. Oxidation Energy Supply and Energy Deposition

Compared to the CON group, the TES group’s OXFAT exhibited an increase (Table 4, *p* < 0.05) and had no effect on OXPRO and OXCHO, but OXPRO in the TES group was lower than that in the CON group (*p* > 0.05). In terms of energy balance, the fat sedimentation energy and sedimentation energy rate in the TES group were higher than those in the CON group (*p* < 0.05), and the FE was lower (*p* < 0.05). There was no difference in HP or protein sedimentation energy between the two groups, but CO_2_ production in the TES was lower than that in the CON group (*p* > 0.05).

### 3.4. Serum Biochemical Indicators

Compared to the CON group, the serum TG of the pigs in the TES group was higher by 32.56% (Table 5, *p* < 0.01), and the serum HDL in the TES group was higher than that in the CON group (*p* < 0.05). There was no difference in TP or GLU between the TES and CON groups, but there was an increasing trend (*p* > 0.05). There were no differences in the serum BUN, LDL, AST, and ALT between the two groups (*p* > 0.05).

### 3.5. Slaughter Performance

Compared to the CON group, the carcass weight, pre-slaughter live weight, backfat thickness, and eye muscle area in the TES group were higher (Table 6, *p* < 0.05), and the body fat content and body fat percentage in the TES group increased by 51.85% and 44.30% (*p* < 0.05). Compared to the CON group, the dressing percentage and lean meat content in the TES group increased, but with no significant difference (*p* > 0.05), and the lean meat percentage between the two groups was not significantly different (*p* > 0.05).

### 3.6. Unit Body Composition and Consumption of Digestible Protein and Energy

To objectively evaluate the efficiency of protein deposition in fattening pigs, we calculated the weight gain, carcass weight, body fat mass, and lean body mass per unit of effective nutrient intake (digestible energy or digestible crude protein). As shown in Table 7, compared to the CON group, the lean meat content/DCP of the TES group increased by 9.32% (*p* < 0.01), and the body fat content/DCP of the TES group was higher than that of the CON group, which increased by 57.69% (*p* < 0.05). Compared to the CON group, there were no differences in weight gain/DCP, weight gain/DE, carcass weight/DCP, carcass weight/DE, lean meat content/DE, or body fat content/DE in the TES group, but there was an increasing trend (*p* > 0.05).

### 3.7. Meat Quality

Compared to the TES group, there were no differences in the marbling score, pH, drip loss, b*, or cooking loss of the longissimus dorsi in the CON group (Table 8, *p* > 0.05). Compared to the CON group, the shear force of the longissimus dorsi in the TES group was lower by 7.01% (*p* < 0.05), and the L* and a* of the longissimus dorsi in the TES group were higher (*p* < 0.05).

## 4. Discussion

Increasing the ADFI is a way to improve heat production under a low-temperature environment to maintain the growth and development of the body. When cold stress increases, although the ADFI increases, the body is unable to maintain normal growth, perhaps because feed intake tends to be saturated, energy is used to maintain body temperature, and energy for growth and development is limited [28,29,30]. In our feeding experiment, we found that the FCR of the TES group increased during the fattening stage, and it was speculated that differences in the dietary energy level after the addition of fat may have a more obvious effect on the feed conversion efficiency in the fattening stage. However, there was no difference in the growth performance of growing pigs, which may be due to the inability of pigs to effectively utilize fat [31]. It has been reported that adding 5% fat can decrease the ADFI and improve the FCR of 150 kg pigs [32]. But the study by Kil et al. revealed that the ADFI of fattening pigs after the addition of 8% soybean oil decreased [33], and the difference in FCR during the fattening stage was greater; this may be because OXFAT is higher than OXCHO, the addition of fat can concentrate energy within a certain range, and eating a slightly smaller diet can enable the body’s energy needs to be met. Ramos-Canahe et al. reported that increasing the dietary energy level by 7.16% improved the growth performance of 10–25 kg Mexican hairless pigs [34]. This study revealed that the consumption of protein energy decreased with increasing dietary fat, which also possibly reduced the absorption of glucose and fatty acids, and the ADFI and FCR in the TES group decreased, indicating that the optimized diet had an effect. However, the differences in dietary energy and fat levels at different stages need further experimental verification.

The metabolizable requirements of animals can increase under a low-temperature environment [35]. The increase in the energy digestibility of the TES group was due to polyunsaturated fatty acid. Powles et al. reported that the addition of soybean oil improved energy digestibility, which was consistent with the results of this study [36]. The energy and nutrient digestibility in the TES group was higher than that in the CON group; this is because the addition of soybean oil led to a decrease in the gastric emptying rate [37] and the digesta passage rate [38]. Fat from added oil is more digestible than fat in feed ingredients, and the negative influence of endogenous losses on fat digestibility is greater at low levels than at high levels of dietary fat, so fat digestibility increased with increasing dietary fat [39], which was similar to the results of Kil et al. [40]. It has been reported that decreasing dietary fat can increase the energy absorbed by the foregut and reduce the energy absorbed by the hindgut [41]. Additionally, dietary fat can restrain the de novo synthesis of fatty acids [42], which may be because the fat part in the feed can be directly deposited into body fat, thus eliminating the process of conversion from carbohydrates to fat and reducing energy consumption. In terms of nitrogen balance, the results of this study showed that the TES group had high protein demand and their nitrogen deposition rate increased due to the increase in the dietary amino acid deposition efficiency of tissue protein synthesis [43], and the ingested energy sources were converted into each other, which improved protein digestibility and decreased FN, indicating that body fat and protein metabolism are affected by dietary nutrient proportions. However, whether the addition of fat to the diet leads to an increase in nitrogen emissions remains to be demonstrated in future experiments.

Our research showed that improving dietary energy levels by adding fat had an effect on the oxidation and energy supply of dietary substances under low temperature environment. The three major energy sources and their metabolites use acetyl-CoA as the hinge, and the process of mutual conversion is accompanied by the absorption and release of energy. Therefore, it is very important to optimize the dietary ratio of energy sources to improve the energy utilization efficiency and improve the growth and physiological state of pigs. Our results showed that the TES group exhibited decreased nutrient intake and substrate oxidation, thereby reducing O_2_ consumption and CO_2_ emissions; the CO_2_ released per unit of fat oxidation was greater than that of carbohydrates, and the results of the slaughter tests showed an increase in fat deposition, which indirectly reduces greenhouse gas emissions. The body is oxidized for carbohydrates alone, and the body cannot meet its energy needs when the ambient temperature decreases, so fat and protein are oxidized for energy [21]; this also corresponds to an RQ of less than 1. The oxidative heat production contribution ratios of OXCHO/OXFAT/OXPRO were 91.11%: 0.57%: 8.32% (CON) and 81.19%: 11.23%: 7.58% (TES), respectively. The OXPRO was reduced, which indicates a connection with UN. The protein deposition was relatively stable in relation to the lean content estimated in the slaughter test. Compared with OXCHO, OXFAT requires more O_2_ and produces less CO_2_, and increased UN in the TES group may indicate that fewer amino acids were directly involved in oxidation and that protein synthesis had higher energy utilization efficiency than fat synthesis when the daily energy intake of pigs was relatively sufficient [44]. The results of this study showed that the TES group exhibited not only reduced CO_2_ production but also accelerated body metabolism, and more energy was used to produce heat to maintain body temperature. The sedimentation energy of the diet and the growth and development of pigs in the TES group increased under the condition of not restricting the intake of energy sources, which was consistent with the above results. Therefore, improving dietary energy levels by adding fat can decrease fattening pigs’ energy loss under a low-temperature environment.

Blood is an important part of the internal environment and can reflect the health status of a pig herd, and blood biochemical indicators can be used to measure the nutritional metabolism of a pig’s body. Metabolic raw materials and waste products are transported through the bloodstream, and changes in the composition of these components directly affect the body’s metabolism [45]. TP is a direct reflection of the body’s protein metabolism [46]. GLU is a direct reflection of the body’s carbohydrate metabolism state [47]. TG, HDL, and LDL are all important indicators of the body’s ability to metabolize lipids [48]. AST and ALT are important indicators of liver metabolism and directly reflect the health status of the body [49]. The optimization of dietary energy had an effect on the serum biochemical indicators in pigs under a low-temperature environment, and the TG, TP, and GLU increased, which was consistent with the results of Wei et al. [30] and could explain why the growth performance in the TES group was higher than that in the CON group. The main reasons for the elevated serum GLU in the high-fat and -energy group may be related to body fat and lean meat, as well as the fact that the glycerol and fatty acids required for TG synthesis are produced mainly by GLU [50]. This study showed that the dietary energy structure and level had no effect on AST or ALT, but these values in the TES group were higher than in the CON group. We found that the energy-optimized diet had no negative impact on serum biochemical indicators and could also improve protein or fat metabolism to within the normal range, which was one of the main reasons for the decrease in FCR and the higher FBW; this could encourage commercial swine producers to apply these strategies and products.

Because pigs need a thicker fat layer to resist the cold, our research showed that the body fat content of the TES group was higher than that of the CON group and the carcass production was higher, which may be due to the reduction in dietary fiber resulting in lower internal organ weight [51]. In general, the lean meat rate of pigs can reflect the body protein deposition, while the body fat percentage mainly reflects the body fat deposition [52], and an increase in fat deposition is conducive to adapting better under a low-temperature environment. For further in-depth research, we calculated the effective product produced per unit of DCP (digestible crude protein) and DE (digestible energy) consumed. We found that for a consumed DCP of 1 kg, the weight gain, carcass weight, body fat content, and lean meat content of the pigs in the TES group increased compared with those in the CON group. At the same time, the consumption of DE of 1 MJ increased the weight gain, carcass weight, body fat content, and lean meat content of the pigs in the TES group compared with those in the CON group. These results proved that the effective product produced by the consumption of protein or energy per unit mass in the TES group was higher than that in the CON group. In short, improving dietary energy levels by adding fat could increase the protein utilization efficiency and reduce heat gain, burn fat for energy, and reduce protein resource consumption under a low-temperature environment.

A cold environment reduces meat color and pH [53], and whether the meat quality can be improved by adjusting dietary energy source distribution and levels needs to be further studied. Related studies have shown that adding kapok seed oil can improve the muscle tenderness of pigs [54], and the shear force of the TES group was lower than that of the CON group, which is consistent with the above results. Lee et al. [55] noted that an increase in dietary energy levels could increase the hydraulicism of muscles, and our results showed that drip loss decreased and that cooking loss was negatively correlated with energy level, indicating that improving dietary energy level by adding fat within a certain range can improve meat quality under a low-temperature environment.

## 5. Conclusions

In conclusion, improving dietary energy levels by adding fat affected the protein and energy utilization of fattening pigs under a low-temperature environment. The appropriate addition of fat to increase dietary energy levels not only improves feed conversion efficiency and affects the calorific ratio of the three nutrients, but also reduces fecal nitrogen and urine nitrogen emissions. In addition, it improves protein deposition efficiency and reduces CO_2_ emissions, which is conducive to saving protein resources and provides a scientific basis for appropriate nutrition for pigs in cold areas.

## Figures and Tables

**Table 1:** Composition and nutrient levels of the experimental diet (air dry basis).

Items	30 to 60 kg Weight Stage		60 to 110 kg Weight Stage	
	CON	TES (8%)	CON	TES (8%)
Corn	46.97%	37.59%	58.46%	47.47%
Corn starch	18.50%	19.90%	15.00%	17.97%
Soybean meal	23.24%	24.44%	13.32%	13.89%
Wheat bran	7.35%	8.86%	6.10%	9.99%
Alfalfa meal	0.10%	0.10%	3.56%	1.89%
Soybean oil	0.70%	6.00%	1.00%	6.00%
CaHPO_4_	0.80%	0.80%	0.49%	0.46%
Limestone	0.83%	0.82%	0.85%	0.93%
NaCl	0.25%	0.25%	0.20%	0.20%
Lysine	0.39%	0.37%	0.31%	0.31%
Methionine	0.14%	0.15%	0.06%	0.08%
Threonine	0.14%	0.14%	0.10%	0.11%
Tryptophane	0.02%	0.02%	0.01%	0.02%
Valine	0.07%	0.06%	0.04%	0.18%
Premix ^(1)^	0.50%	0.50%	0.50%	0.50%
Total	100%	100.00%	100.00%	100.00%
Nutrient levels ^(2)^				
Digestible energy (MJ/kg)	14.20	15.34	14.02	15.14
Crude protein (%)	16.90	16.60	13.62	13.86
Digestible crude protein (%)	13.19	13.19	10.06	10.06
Ether extract (%)	3.15	8.09	3.69	8.33
Crude fiber (%)	5.59	4.45	3.73	3.38
Ash (%)	5.09	3.66	3.80	3.52
Tryptophane (%)	0.17	0.17	0.12	0.12
Methionine + cysteine (%)	0.55	0.55	0.40	0.40
Threonine (%)	0.60	0.60	0.45	0.45
Calcium (%)	0.63	0.63	0.56	0.56
Effective phosphorus (%)	0.27	0.27	0.19	0.19

^(1)^ The premix provided the following per kg of diet: VA 1550 IU, VD 3190 IU, VE 18 IU, VK 0.5 mg, choline 0.50 g, vitamin B6 1.5 mg, vitamin B12 15.0 μg, biotin 0.08 mg, folic acid 0.4 mg, nicotinic acid 15 mg, pantothenic acid 10 mg, thiamine 1.6 mg, riboflavin 3 mg, Cu 4.5 mg, I 0.14 mg, Fe 70 mg, Mn 3 mg, Se 0.30 mg, Zn 70 mg. ^(2)^ Crude protein (GB/T6432-2018) [15], ether extract (GB/T6433-2006) [16], crude fiber (GB/T6434-2022) [17], and ash (GB/T212-2008) [18] are measured values, and the rest are calculated values [calculated according to NRC (2012)].

**Table 2:** Effects on the growth performance of growing and fattening pigs.

Items	CON	TES	*p*-Value
30 to 60 kg weight stage			
IBW (kg)	31.77 ± 2.86	30.73 ± 4.21	0.316
FBW (kg)	57.57 ± 4.60	58.25 ± 4.87	0.764
ADFI (kg/d)	1.83 ± 0.01 ^a^	1.74 ± 0.01 ^b^	<0.001
ADG (kg/d)	0.60 ± 0.06	0.64 ± 0.05	0.357
FCR	3.05 ± 0.37	2.72 ± 0.29	0.083
60 to 110 kg weight stage			
IBW (kg)	57.57 ± 4.60	58.25 ± 4.87	0.758
FBW (kg)	105.17 ± 5.08 ^b^	110.67 ± 5.29 ^a^	0.042
ADFI (kg/d)	3.18 ± 0.03 ^a^	3.03 ± 0.01 ^b^	<0.001
ADG (kg/d)	0.85 ± 0.07	0.94 ± 0.11	0.249
FCR	3.74 ± 0.22 ^a^	3.22 ± 0.34 ^b^	0.046

Data in the same column with different superscript letters are significantly different (*p* < 0.05), whereas data with the same superscript letter are not significantly different (*p* > 0.05). IBW: initial body weight; FBW: final body weight; ADFI: average daily feed intake; ADG: average daily gain; FCR: feed/gain ratio.

**Table 3:** Effects on nutrient digestibility and nitrogen deposition of fattening pigs.

Items	CON	TES	*p*-Value
Digestibility coefficients (%)			
EE	70.61 ± 0.59 ^b^	80.82 ± 1.43 ^a^	<0.001
CP	82.57 ± 1.27	83.36 ± 0.56	0.744
Energy	80.07 ± 2.07 ^b^	82.22 ± 2.04 ^a^	0.008
Nitrogen balance (g/d)			
Nitrogen intake	64.06 ± 2.33	60.94 ± 1.88	0.293
FN	11.17 ± 0.37 ^a^	10.14 ± 0.16 ^b^	<0.001
UN	17.24 ± 0.14 ^a^	14.80 ± 0.74 ^b^	0.026
Nitrogen deposition	35.65 ± 0.62	36.00 ± 2.43	0.652
Nitrogen digestibility rate (%)	82.57 ± 1.27	83.36 ± 0.56	0.741
Nitrogen deposition rate (%)	55.65 ± 1.71 ^b^	59.07 ± 4.72 ^a^	0.017

Data in the same column with different superscript letters indicate significant differences (*p* < 0.05), while data with the same superscript letter indicate no significant differences (*p* > 0.05). EE: ether extract; CP: crude protein; FN: fecal nitrogen; UN: urine nitrogen.

**Table 4:** Effects on energy metabolism of fattening pigs.

Items	CON	TES	*p*-Value
CO_2_ production (L/d)	1066.89 ± 16.53	1021.69 ± 15.54	0.311
O_2_ consumption (L/d)	1088.66 ± 14.63	1075.46 ± 17.74	0.692
RQ	0.98 ± 0.02	0.95 ± 0.04	0.102
OXCHO (MJ/d)	21.74 ± 2.04	18.22 ± 2.79	0.054
OXFAT (MJ/d)	0.14 ± 0.01 ^b^	2.52 ± 0.10 ^a^	0.004
OXPRO (MJ/d)	1.98 ± 0.01	1.70 ± 0.14	0.774
Energy balance (MJ/d)			
GE	52.36 ± 2.57	52.74 ± 2.89	0.213
FE	10.44 ± 0.30 ^a^	9.38 ± 0.62 ^b^	<0.001
UE	1.19 ± 0.08	1.01 ± 0.02	0.102
HP	22.87 ± 1.05	22.44 ± 2.24	0.725
Protein sedimentation energy	4.10 ± 0.13	4.14 ± 0.09	0.854
Fat sedimentation energy	13.76 ± 0.13 ^b^	15.77 ± 0.09 ^a^	0.049
Sedimentation energy	17.86 ± 1.13 ^b^	19.91 ± 2.09 ^a^	<0.001
Sedimentation energy rate (%)	34.11 ± 1.28 ^b^	37.75 ± 2.07 ^a^	0.007

Data in the same column with different superscript letters indicate significant differences (*p* < 0.05), while data with the same superscript letter indicate no significant differences (*p* > 0.05). RQ: respiratory quotient; HP: heat production; OXCHO: oxidation of carbohydrates; OXFAT: oxidation of fat; OXPRO: oxidation of protein; GE: gross energy; FE: feces energy; UE: urine energy.

**Table 5:** Effects on the serum biochemical parameters of fattening pigs.

Items	CON	TES	*p*-Value
BUN (mmol/L)	3.90 ± 0.11	3.87 ± 0.51	0.264
TP (g/L)	72.97 ± 2.75	73.79 ± 4.11	0.723
GLU (mmol/L)	5.02 ± 0.73	5.12 ± 0.54	0.362
TG (mmol/L)	0.43 ± 0.06 ^b^	0.57 ± 0.05 ^a^	<0.001
HDL (mmol/L)	0.90 ± 0.12 ^b^	0.98 ± 0.12 ^a^	0.036
LDL (mmol/L)	3.11 ± 0.14	3.10 ± 0.19	0.801
AST (U/L)	27.00 ± 2.21	28.30 ± 3.33	0.294
ALT (U/L)	26.23 ± 2.74	27.03 ± 2.52	0.513

Data in the same column with different superscript letters indicate significant differences (*p* < 0.05), while data with the same superscript letter indicate no significant differences (*p* > 0.05). BUN: blood urea nitrogen; TP: total protein; GLU: glucose; TG: triglyceride; HDL: high-density lipoprotein; LDL: low-density lipoprotein; AST: aspartate aminotransferase; ALT: alanine aminotransferase.

**Table 6:** Effects on the slaughter performance of fattening pigs.

Items	CON	TES	*p*-Value
Carcass weight (kg)	75.00 ± 5.06 ^b^	79.55 ± 5.28 ^a^	0.026
Pre-slaughter live weight (kg)	105.17 ± 5.08 ^b^	110.67 ± 5.29 ^a^	0.044
Dressing percentage (%)	71.31 ± 3.40	71.64 ± 1.41	0.671
Backfat thickness (mm)	13.27 ± 1.91 ^b^	14.20 ± 0.94 ^a^	0.013
Eye muscle area (cm^2^)	42.93 ± 3.12 ^b^	46.29 ± 2.20 ^a^	0.032
Lean meat content (kg)	54.52 ± 3.64	56.95 ± 2.07	0.687
Body fat content (kg)	7.29 ± 1.02 ^b^	11.07 ± 1.04 ^a^	<0.001
Lean meat percentage (%)	51.84 ± 0.76	51.46 ± 0.91	0.169
Body fat percentage (%)	6.93 ± 0.34 ^b^	10.00 ± 0.67 ^a^	0.047

Data in the same column with different superscript letters indicate significant differences (*p* < 0.05), while data with the same superscript letter indicate no significant differences (*p* > 0.05).

**Table 7:** Effects of available nutrients per unit intake on weight gain, carcass weight, lean meat content, and body fat content in pigs.

Items	CON	TES	*p*-Value
Weight gain/DCP (g/g)	25.94 ± 0.06	29.67 ± 0.28	0.564
Weight gain/DE (g/MJ)	20.31 ± 0.08	21.51 ± 0.29	0.616
Carcass weight/DCP (g/g)	26.51 ± 0.40	29.51 ± 0.41	0.063
Carcass weight/DE (g/MJ)	20.82 ± 0.14	21.41 ± 0.91	0.702
Lean meat content/DCP (g/g)	19.32 ± 0.53 ^b^	21.13 ± 0.34 ^a^	0.047
Lean meat content/DE (g/MJ)	15.14 ± 0.91	15.35 ± 0.94	0.691
Body fat content/DCP (g/g)	2.66 ± 0.02 ^b^	4.17 ± 0.09 ^a^	0.028
Body fat content/DE (g/MJ)	2.08 ± 0.04	2.99 ± 0.07	0.380

Data in the same column with different superscript letters indicate significant differences (*p* < 0.05), while data with the same superscript letter indicate no significant differences (*p* > 0.05). DCP: digestible crude protein; DE: digestible energy.

**Table 8:** Effects on the meat quality of fattening pigs.

Items	CON	TES	*p*-Value
pH 24 h	5.57 ± 0.12	5.59 ± 0.07	0.326
Drip loss over 48 h (%)	4.98 ± 0.14	4.85 ± 0.28	0.924
Cooking loss (%)	27.55 ± 1.48	26.90 ± 2.42	0.643
Shear force (N)	39.07 ± 2.57 ^a^	36.51 ± 3.78 ^b^	0.027
Marbling score	3.48 ± 0.34	3.53 ± 0.40	0.672
Objective meat color			
L* 45 min	44.49 ± 2.34 ^b^	46.97 ± 3.85 ^a^	0.018
a* 45 min	5.41 ± 0.47 ^b^	6.08 ± 1.02 ^a^	0.049
b* 45 min	11.83 ± 0.57	10.96 ± 0.16	0.351

Data in the same column with different superscript letters indicate significant differences (*p* < 0.05), while data with the same superscript letter indicate no significant differences (*p* > 0.05).

## Data Availability

The original contributions presented in this study are included in the article; further inquiries can be directed to the corresponding authors.
